# Psychometric study and validation of an abbreviated version of the Left-Wing Authoritarianism Scale (LWA-9) in Chilean university students

**DOI:** 10.3389/fpsyg.2025.1627540

**Published:** 2025-08-29

**Authors:** Gustavo Troncoso-Tejada, José Luis Gálvez-Nieto, Ignacio Norambuena-Paredes, Miguel Galván-Cabello, David González Casas

**Affiliations:** ^1^Programa de Doctorado en Ciencias Sociales, Universidad de La Frontera, Temuco, Chile; ^2^Departamento de Trabajo Social, Universidad de La Frontera, Temuco, Chile; ^3^Departamento de Trabajo Social y Servicios Sociales, Universidad Complutense de Madrid, Madrid, Spain

**Keywords:** authoritarianism, university students, Chile, psychometric study, left-wing authoritarianism

## Abstract

**Introduction:**

Left-wing authoritarianism is a multidimensional construct that is gaining increasing interest. This study evaluated the validity and reliability of three versions of the Left-Wing Authoritarianism Scale (LWA) among Chilean university students and proposed a shortened 9-item version (LWA-9) that significantly reduces the length of the instrument and facilitates its use in applied contexts.

**Methods:**

A non-probabilistic convenience sampling method was used, involving 415 Chilean university students (69.2% women, 30.1% men, and 0.7% identifying as another gender), aged between 17 and 44 years (*M* = 20.06; SD = 3.42). Participants completed a sociodemographic questionnaire, the LWA scale, the Social Dominance Orientation scale (SDO-7), and the Right-Wing Authoritarianism scale (RWA). Exploratory Structural Equation Modeling (ESEM) analyses were conducted to evaluate the factorial structure.

**Results:**

Analyses confirmed a correlated three-factor structure for all versions. The LWA-9 version stood out for better psychometric fit, high factor loadings, robust reliability levels, and factorial invariance by sex up to strict invariance, although it only reached configural invariance by age group. Convergent validity was supported by positive and significant correlations with the SDO-7 dimensions, and discriminant validity by low and non-significant correlations with the RWA scale, indicating that the LWA-9 captures a distinct construct.

**Discussion:**

These results support the structural validity of the LWA in all its versions and its empirical utility as a brief, efficient, and theoretically sound measure of left-wing authoritarianism.

## 1 Introduction

After the mid-20th century, the construct of authoritarianism has been mainly associated with right-wing ideologies (Jankowski et al., 2022). Most psychological studies have focused on understanding the manifestations of authoritarianism in individuals and politically conservative groups (Avendaño et al., 2022). In this sense, these assertions align with the observation of authoritarian individuals based on the authoritarianism model developed by Altemeyer (1981), who characterizes it in three dimensions: authoritarian aggression, which involves intentionally causing harm to others; authoritarian submission, which refers to the voluntary and unquestioning acceptance of the decisions of authorities, fully trusting them; and conventionalism, which denotes support for society’s traditions and norms (Altemeyer, 1981).

Some emerging studies on authoritarianism expand the traditional measure of observation, positioning its study not only from the historical right-wing orientation, but also from a left-wing ideological perspective (Costello and Patrick, 2023; Deverson et al., 2025). These studies suggest a constellation of traits that include prejudice against those who are different, the willingness to exercise authority within a social group to coerce individuals’ behavior, cognitive rigidity, aggression and punishment toward enemies perceived as different, overvaluation of hierarchies and social status, and moral absolutism (Costello et al., 2022; Opongo, 2024). Despite this evidence, the study of authoritarianism from a left-wing ideological perspective remains controversial (Conway et al., 2018; Malecki et al., 2025).

Some authors warn that the right-wing ideological component present in the traditional model could introduce a bias in the analysis of the phenomenon (Avendaño et al., 2022). This is due to the demonstrated existence of authoritarian attitudes in left-wing groups in different parts of the world, both in the United States (Conway et al., 2018; Conway and McFarland, 2019; Federico et al., 2017; Manson, 2020) and in Europe (De Regt et al., 2011; Van Hiel et al., 2006). In addition, they identify its presence in Spanish-speaking contexts (Fasce and Avendaño, 2022) as a consequence of a reactionary drift in left-wing political groups that have adopted rigid and conservative positions in these countries (Ovejero, 2018).

García-Sánchez et al. (2022a,b) points out that the study of authoritarianism in Latin America needs to broaden the debate and emphasizes the need to develop appropriate instruments for its measurement (García-Sánchez et al., 2022a,b). These instruments should consider sociohistorical conditions (Obreque et al., 2024) and overcome the near-universal exclusion of leftist ideological traits present in much of the historical conceptualizations of authoritarianism (Costello et al., 2022; Manson, 2020).

Other studies indicate a concern about the consequences of global unrest, mobilizing social fears and slogans toward exclusionary cultural identifications, violence, and social and institutional disaffection (Feddersen et al., 2024; Stiglitz, 2010; Van de Velde, 2023). These events could encourage a tendency toward authoritarianism in people’s behavior, highlighting the importance of broadening the observation perspective (Nilsson and Jost, 2020).

Research indicates that university students in Chile show high levels of radicalism and violence when identifying with the slogans of social movements (Álvarez et al., 2024; Li et al., 2023; Sánchez-Barría and Miranda, 2022). This highlights the importance of considering other previous findings. These warn that individuals with a stronger identification with social movements may display more willingness to engage in violent radical actions (Da Costa et al., 2023; Kowzan and Szczygiel, 2023). In more recent social movements, it is evident that university students interested in politics, especially those identified with the left, are more participative and tend to justify violent actions as a means to achieve social change (Cox et al., 2024; Gerber et al., 2023).

Among the prominent slogans are the issues of economic inequality affecting the middle class in the country, demands for a fairer society, and structural transformations in the democratic system (Álvarez et al., 2024; Rivera-Aguilera et al., 2021), which have influenced the perception of anomie and may have impacted the demands for greater equality that emerged during the context of the Social Outburst of October 2019, as well as the political fragmentation and growing polarization of current Chilean society (Cea-Leiva et al., 2024; Candia et al., 2021).

From this scenario, the evidence shows that in Chile, these problems of economic and social inequality and dissatisfaction with the democratic system contribute to the emergence of authoritarian components in individuals and groups within society (Castro Ríos, 2012). These feelings of dissatisfaction and powerlessness generate feelings whose impact is crucial for satisfaction in everyday life (Barros-Bustos et al., 2019).

Considering the above, this study evaluated the validity and reliability of the three versions of the Left-Wing Authoritarianism Scale (Costello and Patrick, 2023) among Chilean university students and proposed a shortened 9-item version (LWA-9), which significantly reduces the instrument’s length and facilitates its use in applied contexts. For this purpose, the Spanish abbreviated version of the Left-Wing Authoritarianism Index validated by Avendaño et al. (2022) was used as a basis, as it derives directly from the original English scale (Costello and Patrick, 2023). The version by Avendaño et al. (2022) was selected due to its prior validation in a Spanish-speaking context. This aligns with the theoretical and empirical relevance of broadening the perspective of observation of the authoritarianism construct from a left-wing ideological perspective.

### 1.1 Development of left-wing authoritarianism measurements

Efforts to measure and observe manifestations of authoritarianism have generated extensive study data. Among the initial contributions, the authors of *The Authoritarian Personality* (Adorno et al., 1950) stand out. Supported by psychoanalysis, they sought to identify highly fascist psychological profiles, characterized by antisemitic behavior and prejudice toward others (Stone, 1980). Identifying these profiles proved relevant in the mid-20th century, due to the latent threat to the stability of Western democracies in the European context following World War II. In this regard, fascism was measured through the F Scale, which in its factorial structure includes nine factors (Adorno et al., 2006; Crochik, 2021).

Historically, research on authoritarianism has primarily focused on right-wing ideology (Altemeyer, 1981). However, in an attempt to conceptualize authoritarianism from a left-wing ideological perspective, Altemeyer (1996) described the same factors used for right-wing authoritarianism—aggression, submission, and conventionalism—but directed toward the annulment of authority figures. This interpretation, however, has been considered less studied and lacks sufficient scientific support to demonstrate that the left-wing authoritarianism index possesses the same factorial structures as that of the right (Avendaño et al., 2022).

In continuity, various authors have argued that LWA is a valid and reliable construct (Feldman, 2003; McFarland et al., 1996; Mullen et al., 2003; Ray, 1983; Van Hiel et al., 2006). This has led to testing left-wing authoritarianism scales in specific populations (Van Hiel et al., 2006) and in post-communist countries of Eastern Europe (De Regt et al., 2011). These tests, despite showing internal consistency in their factorial structures, conclude that specificity and context represent limitations for the applicability of an LWA in Spanish-speaking contexts due to the gap in meanings and political-historical events.

On the other hand, recent research supports the symmetry hypothesis, suggesting that authoritarianism manifests in both right-wing and left-wing contexts. Studies with university students have shown similar scores, with significant positive correlations between Left-Wing Authoritarianism (LWA) and measures of liberalism, prejudice, dogmatism, and attitude strength, positioning LWA as a viable construct in U. S. samples (Conway et al., 2018). However, some authors argue that focusing on a one-factor structure for comparative purposes fails to capture the complexity of left-wing authoritarianism (Avendaño et al., 2022), and once again highlights the psychometric issues found in Altemeyer’s measurements (Nilsson and Jost, 2020).

From this scenario, the contributions of Costello and Patrick (2023) are significant to the study of the construct, due to the development of a multidimensional measure comprising three dimensions: anti-hierarchical aggression, which refers to the willingness to punish established structures of authority and power violently; anticonventionalism, understood as a sense of superiority or moral absolutism and a desire for ideological uniformity within a group; and finally, top-down or downward censorship, defined as the motivation to exercise authority and regulate non-leftist behaviors within a group in a coercive, punitive, and ideological manner.

As discussed by Avendaño et al. (2022), these contributions are more comprehensively integrated, and their conceptualization allows for the observation of sociopolitical landscapes within the context of Latin America and among Spanish-speaking populations. Likewise, the Left-Wing Authoritarianism Scale (Costello and Patrick, 2023) is presented as a tool to analyze and understand illiberal agendas occurring within left-wing political parties and movements in countries outside the United States (Campbell and Manning, 2018; Lukianoff and Haidt, 2019). From a perspective contextualized to the Chilean reality, this measure could broaden the view regarding the manifestations of authoritarianism in its authoritarian tendencies, anti-democratic practices, intolerance toward opposing opinions, freedom of expression, etc. This would allow a deeper understanding of how authoritarianism can manifest from a left-wing ideological orientation.

The contributions of LWA could predict key psychological and behavioral outcomes related to the acceptance of violence. In addition, the three correlated factors offer a broad and accurate view of authoritarian dominance, facilitating a deeper understanding of the core of Social Dominance Orientation (SDO). At the same time, the nomological network of LWA is similar to that of SDO (Costello et al., 2022). This dimension reflects both authoritarian submission and dominance (Asbrock et al., 2010; Duckitt and Sibley, 2007) and has traditionally been associated with right-wing ideological profiles, that is, among the dimensions of RWA and SDO (Costello et al., 2022; Osborne et al., 2023).

Concerning the above, SDO describes support for hierarchy and the domination of powerful groups over weaker ones, while RWA describes support for coercive social control and obedience to authority (García-Sánchez et al., 2022b; Ho et al., 2015; Obreque et al., 2024). In this regard, LWA, within its nomological structures, reflects motivations to overthrow the established hierarchy and punish those in power forcibly. This could offer new theoretical and conceptual explanations regarding the defining core of SDO (Costello et al., 2022; Milfont and Osborne, 2024; Peng, 2022).

The empirical results show that the relationship between left-wing authoritarianism (LWA) and social dominance orientation (SDO) varies depending on the context and the sample analyzed. For example, Peng (2022), in a U. S. sample, reported a non-significant correlation between the two constructs (*r* = 0.05), indicating a possible independence between them in that context. However, more detailed studies that disaggregate the constructs into specific factors offer a more nuanced perspective.

Milfont and Osborne (2024) examined the correlations between LWA factors—Antihierarchical Aggression (AHA), Anti-Conventionalism (AC), and Top-Down Censorship (TDC)—and SDO factors—Dominance (SDO-D) and Anti-Egalitarianism (SDO-E)—in samples from the United States (*n* = 512) and New Zealand (*n* = 447). In the United States, correlations between these factors ranged from *r* = −0.08 to *r* = −0.59, with most being significant (*p* < 0.001), except for the correlation between LWA-AHA and SDO-D, which was not significant. In contrast, in New Zealand, a positive and significant correlation was observed between LWA-AHA and SDO-D (*r* = 0.34), while the relationship between LWA-AC and SDO-D was positive but not significant (*r* = 0.07). The remaining correlations in this sample were negative, ranging from *r* = −0.06 to *r* = −0.38, all of which were significant except for LWA-TDC and SDO-D. These results reflect that the relationships between LWA and SDO factors can vary in direction and magnitude depending on cultural context and the internal structure of the constructs.

The study by Van Hiel et al. (2024), conducted with a sample of adolescents, revealed a positive and significant relationship between LWA and SDO. However, the magnitude of this relationship varied depending on the type of analysis. Bivariate correlations showed a moderate association (*r* = 0.22, *p* < 0.001), which increased slightly when controlling for age and sex (*r* = 0.25, *p* < 0.001), and the structural equation modeling analysis yielded a more robust correlation between the latent factors (*r* = 0.39; 95% CI: [0.28, 0.50]). These findings demonstrate that the relationship between LWA and SDO is sensitive to contextual, age-related, and cultural factors, and that the links between authoritarianism and social dominance can manifest differently across the political spectrum and throughout different stages of life.

The Chilean context offers unique perspectives on this relationship, particularly within mobilized university populations. The study by Obreque et al. (2024) with Chilean students (*n* = 341) shows significant correlations between RWA and SDO dimensions (*r* = 0.57 for group dominance; *r* = 0.32 for opposition to equality), establishing a suggestive analytical parallel for future research on LWA. In this regard, the empirical contributions of Obreque et al. (2024) broaden the scope of observation on how anti-hierarchical aggression and top-down censorship—factors present in the LWA construct—could be linked with social dominance mechanisms in progressive movements, particularly in scenarios of high political conflict where demands for structural change coexist with vertical practices of internal organization (Costello et al., 2022). In this sense, the Chilean case, characterized in recent decades by student mobilization cycles and social justice demands, offers a natural laboratory for examining these dynamics within non-traditional ideological profiles. Although LWA and RWA are conceptually distinct constructs, reflecting different ideological orientations and motivational bases, their interrelations with mechanisms of social dominance suggest important avenues for comparative research, and the Chilean context provides fertile ground for exploring these dynamics in future studies.

The linguistic and psychometric adaptation of the Left-Wing Authoritarianism (LWA) scale in the Chilean context is essential to ensure the validity and relevance of the instrument. Although the original version had previously been adapted into Spanish (Avendaño et al., 2022), the present study deemed it necessary to conduct a complementary linguistic evaluation to identify and correct potential variations arising from cultural and contextual differences specific to Chile. As noted by Muñiz et al. (2013), sharing the same language does not guarantee semantic or cultural equivalence among Spanish-speaking countries, since local idioms and contextual references can influence the comprehension and interpretation of items (Freiberg-Hoffmann et al., 2022). In this regard, the linguistic and cultural review conducted ensures the clarity and appropriateness of the items for the Chilean population.

In scale validation, it is essential to progressively assess configural, metric, scalar, and strict invariance in order to ensure the comparability of results across groups (Vandenberg and Lance, 2000; Chen, 2007). In this regard, Lambert et al. (2024) confirmed that the Left-Wing Authoritarianism Index-13 (LWAI-13) demonstrates scalar invariance with respect to sex and age in a representative sample of U. S. adults, which supports the validity of comparisons across sexes and generations in future studies. These findings suggest that the LWAI-13 assesses the construct of left-wing authoritarianism equivalently across different demographic subgroups.

Recent literature suggests that little attention has been given to measuring factorial invariance, as is often the case in most psychological research (Nilsson, 2024; Flake et al., 2017), highlighting the need to expand evaluations in this area. Although the study by Krispenz et al. (2025) did not report measurement by age and sex, it did find configural, metric, and scalar invariance across countries for the abbreviated versions of the Left-Wing Authoritarianism Scale in samples from Austria, Germany, and Switzerland. For their part, Jami and Kemmelmeier (2025) demonstrated that in the validation of their Left-Wing Populist Attitudes (LWP) scale—supported by the LWA as a criterion variable—scalar invariance was achieved between men and women, but not by age. In this regard, our study highlights the relevance of expanding the literature by incorporating the analysis of factorial invariance as a central component in the research and application of the Left-Wing Authoritarianism Scale in the Chilean population, emphasizing the importance of focusing on invariance measurement (Nilsson, 2023) and addressing a gap that other works on LWA have not systematically explored.

Given this background and the absence of validated instruments to measure left-wing authoritarianism in the local context, the present study had two objectives: to evaluate the validity and reliability of three versions of the Left-Wing Authoritarianism Scale in Chilean university students and to propose a shortened 9-item version (LWA-9), which significantly reduces the length of the instrument and facilitates its use in applied contexts. The scale used was initially developed in English by Costello and Patrick (2023) and later adapted into Spanish in Spain by Avendaño et al. (2022), allowing for its comparative use and adaptation in Spanish-speaking contexts. In this way, the present work aims to contribute to the literature by providing evidence on the validity and reliability of a shortened version of the scale, suitable for the Chilean context and relevant for future studies in the region.

## 2 Materials and methods

### 2.1 Participants

The participants were selected through non-probability sampling, forming a sample of 415 university students (69.2% women, 30.1% men, and 0.7% of another gender), aged 17–44 years (*M* = 20.06, Sd = 3.42). These students come from various fields of study, mainly: Social Sciences (87.7%), Engineering (10.4%), and Health (1.9%). Regarding their civic-social orientation, the majority of students reported not identifying with any political position (44.4%), followed by those who identified with center-left and left-wing positions (34.7%), center-right and right-wing (15.6%), and finally, with the center (5.3%). Other characteristics are described in Table 1.

**Table 1:** Sociodemographic characterization of the sample.

Variables	Categories	*n* (%)
Age	17–21	82.2%
	22–26	14.5%
	27–31	1.4%
	32–44	1.9%
Nationality	Chilean	99.1%
	Argentinian	0.2%
	Colombian	0.2%
	Venezuelan	0.5%
Residence	Urban	80%
	Rural	20%
Ethnic group	No	67.2%
	Yes	32.8%

### 2.2 Instruments

Three instruments were used in this study: a sociodemographic questionnaire, the Left-Wing Authoritarianism Scale (LWA-39), the Social Dominance Orientation Scale (SDO7), and Right Wing Authoritarianism Scale (RWA).

A sociodemographic questionnaire was first applied with closed-ended questions such as: age, sex, gender, family background (urban/rural), ethnicity, sexual orientation, civic-social orientation, university, degree program, and year of study. Este instrumento permitió recoger información básica sobre los participantes. Su propósito fue caracterizar la muestra y permitir el análisis de posibles diferencias sociodemográficas en las variables del estudio.

Left-Wing Authoritarianism Index LWA (Costello and Patrick, 2023) is a 39-item self-report scale answered using a 7-point ordinal response scale (1 = strongly disapprove, 7 = strongly approve). The LWA presents a factorial structure of three correlated factors named: Anti-Hierarchical Aggression (13 items, e.g., “1. The rich should be stripped of their belongings and status.”), Top-Down Censorship (13 items, e.g., “15. Anyone who opposes gay marriage must be homophobic.”), Moreover, Anti-Conventionalism (13 items, e.g., “33. I am in favor of allowing the government to shut down right-wing internet sites and blogs that promote nutty, hateful positions.”). This scale showed evidence of validity supporting the structure of three correlated factors in its application to two samples. Sample 1 had a size of *n* = 834 (CFI = 0.96; TLI = 0.96; RMSEA = 0.075; SRMR = 0.047). Sample 2 had a size of *n* = 477 (CFI = 0.96; TLI = 0.97; RMSEA = 0.068; SRMR = 0.047). In addition, it demonstrated acceptable levels of internal consistency: Anti-Hierarchical Aggression *α* = 0.91, Top-Down Censorship α = 0.88, and Anti-Conventionalism *α* = 0.92. Its purpose in this study was to assess the multidimensional construct of left-wing authoritarianism in Chilean university students and to analyze the psychometric properties of both the full and abbreviated versions.

The Social Dominance Orientation Scale SDO7 (Ho et al., 2015) was used. This self-report instrument consists of 16 items answered using a 7-point ordinal response scale (1 = strongly oppose, 7 = strongly favor). The SDO7 presents a factorial structure of two correlated factors named: Dominance (8 items, e.g., “1. Some groups of people must be kept in their place.”) and Anti-Egalitarianism (8 items, e.g., “9. We should not push for group equality.”). This scale distinguishes between the subscales of Dominance (SDO-D) and Anti-egalitarianism (SDO-E). In Sample 1 (*n* = 528), the two-factor model showed acceptable fit (CFI = 0.98; RMSEA = 0.08; *χ*^2^/df = 4.08). Additionally, the SDO7 scale demonstrated adequate levels of internal consistency, with *α* = 0.86 for the Dominance (SDO-D) subscale and *α* = 0.87 for the Anti-egalitarianism (SDO-E) subscale. Its purpose in this study was to assess the convergent validity of the LWA, considering the theoretical and empirical relationship between left-wing authoritarianism and social dominance orientation.

The Right-Wing Authoritarianism Scale (RWA; García-Sánchez et al., 2022a,b) was used. It is an 11-item self-report scale designed to assess right-wing authoritarianism in Spanish-speaking contexts. Items are answered on a 7-point ordinal scale (1 = strongly disagree, 7 = strongly agree). The RWA distinguishes three correlated factors: Authoritarian Aggression (e.g., “Our country needs a strong leader to crush the extremists and immoral people who are prevalent in our society.”), Authoritarian Submission (e.g., “Society needs to be more open to people who think differently rather than support a strong leader.”), and Conventionalism (e.g., “Traditional customs and values remain the best way to live.”). The study of the RWA scale in a Spanish-speaking population showed adequate fit indices in confirmatory factor analysis (*n* = 396: CFI = 0.943; TLI = 0.919; RMSEA = 0.045). Additionally, it demonstrated acceptable levels of internal consistency for the overall RWA scale (*α* = 0.63), and for each factor: Authoritarian Aggression (*α* = 0.62), Authoritarian Submission (*α* = 0.61), and Conventionalism (*α* = 0.47).

### 2.3 Procedures

The study was approved by the Scientific Ethics Committee of the University of La Frontera (record UFRO No. 021_25). To administer the instrument, contact was made with the authorities of the participating universities, who granted institutional consent. Following the methodological criteria of Muñiz et al. (2013), a panel of expert judges conducted a linguistic review of the scale items to adapt colloquial expressions and ensure comprehension by the target population.

Data was collected through a digital questionnaire on the QuestionPro platform. The research team distributed invitations to participants via email, including the questionnaire link and the informed consent form. This document explained the study’s objectives, the voluntary nature of participation, the confidentiality and anonymity of the information, and the right to withdraw at any time.

Participants responded to three instruments: a sociodemographic questionnaire, the Left-Wing Authoritarianism Scale (LWA-39), and the Social Dominance Orientation Scale (SDO7). After data collection, the database was cleaned by removing incomplete records, duplicate responses, and those with implausible response patterns. Only cases with complete data on the main variables were included in the analysis.

For the development of the abbreviated version of the LWA scale, a subset of items was selected based on two criteria: (a) high statistical performance, reflected in strong factor loadings, good correlations with the total scale, and wide variability in responses; and (b) conceptual coherence, ensuring high face validity between item and dimension (Stanton et al., 2002).

### 2.4 Data analysis

The data analysis was carried out in several successive stages. First, descriptive statistics (mean, standard deviation, skewness, and kurtosis) were calculated for the 39 original items of the Left-Wing Authoritarianism (LWA) scale, using the SPSS v.25 software.

Subsequently, the factorial structure of different abbreviated versions of the scale (LWA-39, LWA-25, LWA-13, and LWA-9) was evaluated using Confirmatory Factor Analysis (CFA) models and Exploratory Structural Equation Modeling (ESEM), through the Mplus v.8.1 software (Muthén and Muthén, 2017). For the ESEM estimations, geomin rotation and the WLSMV estimator were used, which is appropriate for ordinal variables and non-normality (Asparouhov and Muthén, 2009). The methodological decision to incorporate ESEM was based on the multidimensional and correlated nature of the construct, as well as the possibility of cross-loadings that traditional CFA might not adequately capture.

The comparison of factorial models was based on the following fit indices: chi-square (*χ*^2^), Comparative Fit Index (CFI), Tucker-Lewis Index (TLI), and Root Mean Square Error of Approximation (RMSEA). Following the recommendations of Browne and Cudeck (1992), values of CFI and TLI greater than or equal to 0.90 and RMSEA values less than or equal to 0.08 were considered indicators of good fit.

Based on the psychometric performance observed in the previous versions, a shortened 9-item version (LWA-9) was developed by selecting three items per theoretical dimension (Anti-Hierarchical Aggression, Anti-Conventionalism, and Top-Down Censorship). The selection was based on a combination of theoretical criteria, such as conceptual representativeness, and empirical criteria, including factor loading, standard error, and contribution to the overall model fit.

Subsequently, CFA and ESEM models were estimated for the LWA-9, comparing their fit with the more extended versions. In addition, multigroup factorial invariance by sex and age group was assessed using a sequential strategy, involving the configural model (M0), metric model (M1), scalar model (M2), and strict model (M3) (Vandenberg and Lance, 2000). Evidence of invariance was based on the criteria proposed by Chen (2007), which considered changes in fit indices (ΔCFI values of 0.01 or less and ΔRMSEA values of 0.015 or less).

The convergent validity of the LWA-9 version was evaluated through correlations with the dimensions of the Social Dominance Orientation-7 (SDO7), expecting positive associations with left-wing authoritarianism, particularly with the anti-egalitarianism dimension.

Finally, the reliability of the factors of the LWA-9 version was estimated using Cronbach’s *α*, McDonald’s *ω*, and GLB coefficients, through the JASP v.0.12.2 software (Trizano-Hermosilla et al., 2021). All analyses were conducted using a confirmatory approach, with interpretations aligned with the construct’s theoretical framework.

## 3 Results

### 3.1 Descriptive analysis

The sample consisted of 415 Chilean university students (69.2% women, 30.1% men, and 0.7% identifying with other genders), aged between 17 and 44 years (*M* = 20.06; SD = 3.42), as detailed in the Participants section (Table 1). This distribution aligns with the national trend in undergraduate enrollment in Chile, where women represent 53.2% and men 46.8% (Higher Education Information Service [SIES], 2024). Similarly, the concentration of participants aged 17–24 reflects the predominant demographic profile in Chilean higher education, as younger age groups have shown the greatest growth in enrollment in recent years.

Table 2 presents the main descriptive and psychometric statistics for the 39 LWA items. The items with the highest means were item 28 (*M* = 5.77; SD = 1.34) and item 37 (*M* = 5.40; SD = 1.73), while the lowest means were for item 2 (*M* = 2.31; SD = 1.36) and item 1 (*M* = 2.76; SD = 1.50). Similarly, the items with the highest skewness were items 2 (skewness = 1.17) and 11 (skewness = 0.92), indicating a distribution skewed toward lower values. Likewise, the highest kurtosis values were for items 2 (kurtosis = 1.13) and 28 (kurtosis = 0.89), showing a greater response concentration than a normal distribution.

**Table 2:** Descriptive statistics and psychometric indicators for the items of the Left-Wing Authoritarianism Scale (LWA).

Descriptive statistics					ESEM LWA-39		
Items	Mean	Sd	Skewness	Kurtosis	AHA	AC	TDC
It1	2.76	1.50	0.69	−0.14	0.900**	0.026 ns	0.098 ns
It2	2.31	1.36	1.17	1.13	0.914**	−0.010 ns	0.091 ns
It3	2.77	1.50	0.67	−0.24	0.783**	0.056 ns	0.030 ns
It4	2.71	1.44	0.70	−0.03	0.715**	0.138*	−0.011 ns
It5	2.64	1.68	0.83	−0.25	0.351**	0.403**	−0.240**
It6	3.73	1.75	0.05	−0.93	0.360**	0.478**	0.030 ns
It7	3.07	1.58	0.46	−0.46	0.473**	0.253**	−0.074 ns
It8	2.96	1.49	0.45	−0.43	0.254**	0.021 ns	−0.152*
It9	3.48	1.53	0.01	−0.62	0.398**	0.228**	−0.066 ns
It10	3.34	1.64	0.20	−0.74	0.287**	0.164**	−0.147*
It11	2.74	1.74	0.92	−0.04	0.264**	0.360**	−0.135*
It12	2.81	1.76	0.70	−0.52	0.261**	0.505**	−0.225*
It13	2.66	1.55	0.56	−0.44	0.364**	0.342**	−0.064 ns
It14	5.41	1.67	−1.04	0.43	−0.042 ns	0.624**	0.107*
It15	4.69	2.02	−0.45	−1.01	−0.216*	0.813**	0.070 ns
It16	3.59	1.88	0.15	−1.05	0.075 ns	0.760**	0.010 ns
It17	4.02	1.78	−0.16	−0.75	0.101*	0.791**	0.006 ns
It18	3.69	1.68	0.04	−0.69	0.037 ns	0.745**	0.055 ns
It19	3.73	1.28	−0.54	0.33	0.113*	0.396**	−0.027 ns
It20	3.28	1.86	0.34	−0.87	0.019 ns	0.813**	−0.205**
It21	2.84	1.77	0.72	−0.37	−0.019 ns	0.782**	−0.228**
It22	2.68	1.59	0.52	−0.62	0.115*	0.675**	−0.266**
It23	3.76	1.55	−0.13	−0.24	0.127*	0.630**	−0.019 ns
It24	4.41	1.73	−0.26	−0.56	−0.101 ns	0.761**	0.012 ns
It25	3.54	1.74	0.16	−0.78	−0.181*	0.673**	−0.085 ns
It26	4.04	1.79	−0.06	−0.84	−0.088 ns	0.839**	−0.047 ns
It27	5.10	1.59	−0.58	−0.35	−0.035 ns	0.147*	0.418**
It28	5.77	1.34	−1.10	0.89	−0.068 ns	0.221**	0.597**
It29	4.88	1.50	−0.49	0.01	0.329**	−0.005 ns	0.690**
It30	4.51	1.58	−0.12	−0.56	0.383**	−0.014 ns	0.477**
It31	5.39	1.25	−0.55	0.22	0.029 ns	0.183**	0.564**
It32	3.19	1.74	0.36	−0.81	0.221**	0.308**	0.012 ns
It33	4.63	1.86	−0.46	−0.76	0.006 ns	0.280**	0.652**
It34	3.95	1.58	−0.16	−0.46	0.021 ns	0.153**	0.606**
It35	4.27	1.66	−0.24	−0.51	0.161*	0.489**	0.178**
It36	3.99	1.72	−0.01	−0.67	0.082 ns	0.231**	0.664**
It37	5.40	1.73	−1.01	0.14	0.051 ns	0.431**	0.530**
It38	5.16	1.83	−0.86	−0.21	−0.108 ns	0.587**	0.493**
It39	5.03	1.73	−0.53	−0.56	−0.057 ns	0.640**	0.307**

AHA, anti-hierarchical aggression; AC, anti-conventionalism; TDC, top-down censorship. ESEM, exploratory structural equation modeling. Sd, standard deviation. Factor loadings were estimated using ESEM. Bolded items correspond to the abbreviated version of the LWA scale (LWA-9). **p < 0.001, *p < 0.05; ns, not significant.

### 3.2 Evaluation of the factorial structure: comparison of LWA versions

First, the factorial structure of different versions of the Left-Wing Authoritarianism (LWA) scale was evaluated, starting with the original 39-item version (LWA-39) (Costello and Patrick, 2023) and continuing with the 25- and 13-item versions previously used in the literature (Table 3). For each version, factorial models were estimated using both Confirmatory Factor Analysis (CFA) and Exploratory Structural Equation Modeling (ESEM), considering that the sample size used in this study (*n* = 415) exceeds the minimum recommended for these analyses (Kline, 2016).

**Table 3:** Comparison of fit between CFA (M0) and ESEM (M1) models across LWA versions.

Versions	Model	WLSMV-*χ*^2^ (df)	CFI	TLI	RMSEA (90 C. I.)
LWA-39	M0	2264.239 (699)	0.863	0.855	0.072 (0.068–0.075)
LWA-39	M1	1634.865 (627)	0.946	0.937	0.061 (0.057–0.064)
LWA-25	M0	2108.924 (272)	0.844	0.828	0.124 (0.119–0.129)
LWA-25	M1	821.747 (228)	0.950	0.934	0.077 (0.070–0.080)
LWA-13	M0	528.880 (62)	0.889	0.861	0.131 (0.121–0.142)
LWA-13	M1	98.666 (42)	0.987	0.975	0.056 (0.041–0.070)
LWA-9	M0	59.080 (24)	0.994	0.991	0.058 (0.039–0.077)
LWA-9	M1	24.896 (12)	0.998	0.993	0.050 (0.021–0.077)

M0, confirmatory factor analysis model (CFA); M1, exploratory structural equation modeling (ESEM); WLSMV-χ^2^, chi-square statistic estimated using the Weighted Least Squares Mean and Variance adjusted estimator (WLSMV); df, degrees of freedom; CFI, comparative fit index; TLI, Tucker–Lewis Index; RMSEA, Root Mean Square Error of Approximation; 90 C. I., 90% confidence interval for RMSEA.

The use of ESEM was methodologically justified considering that scales like the LWA, with theoretically related dimensions and potential cross-loadings, can benefit from a more flexible estimation than that offered by traditional CFA. This decision is based on previous studies that validated the scale in its original version (Costello and Patrick, 2023) and international adaptations (Avendaño et al., 2022).

The results showed that all ESEM models evaluated for LWA-39, LWA-25, and LWA-13 presented satisfactory fit indices. However, when comparing the models, the best psychometric fit was observed in the ESEM model of the 9-item version (LWA-9). This suggests that, in addition to its efficiency in terms of length, the shortened version offers a solid factorial representation consistent with the underlying theory.

### 3.3 Development and validation of the shortened LWA-9 version

The LWA-9 version was developed using a strategy that combined theoretical and empirical criteria. First, the individual statistical performance of the items in the previous factorial models (ESEM and CFA) was reviewed, considering factor loadings, standard errors, and contribution to the overall model fit. Second, the theoretical relevance of each item was assessed in relation to the three dimensions of the construct: Anti-Hierarchical Aggression, Anti-Conventionalism, and Top-Down Censorship.

Based on these criteria, three items per factor were selected, prioritizing those with greater conceptual clarity and robust psychometric performance. This decision is grounded in the need to develop a concise yet conceptually balanced scale, which maintains coverage of the original factors and facilitates its use in applied research contexts.

Finally, an ESEM and a CFA model were estimated for the LWA-9. These models exhibited excellent fit indices (Table 4), characterized by high and significant factor loadings, as well as moderate correlations among the factors. The results empirically support the structural validity of the shortened version, offering a psychometrically sound and operationally more efficient alternative compared to the more extended versions.

**Table 4:** LWA-9 factor structure.

Factors/items	Factor loadings	Standard error	Est./S. E.	*p*-value
Anti-hierarchical aggression by				
It1	0.927	0.012	77.465	*p* < 0.001
It2	0.912	0.014	67.013	*p* < 0.001
It3	0.737	0.022	33.164	*p* < 0.001
Anti-conventionalism by				
It16	0.823	0.018	44.751	*p* < 0.001
It17	0.858	0.016	53.978	*p* < 0.001
It20	0.830	0.018	46.723	*p* < 0.001
Top-down censorship by				
It33	0.728	0.028	25.937	*p* < 0.001
It34	0.654	0.032	20.397	*p* < 0.001
It36	0.798	0.025	32.427	*p* < 0.001

Correlation between factors	Estimate	S. E.	Est./S. E.	*p*-value
Anti-conventionalism with				
Anti-hierarchical aggression	0.653	0.031	20.794	*p* < 0.001
Top-down censorship with				
Anti-hierarchical aggression	0.538	0.040	13.564	*p* < 0.001
Anti-conventionalism	0.847	0.022	39.097	*p* < 0.001

Est./S. E., estimate divided by standard error.

### 3.4 Factorial invariance

A multigroup factorial invariance analysis was conducted to assess the measurement equivalence by sex and age group of the participants (Table 5). First, the configural invariance model (M0) was examined, yielding adequate fit indices in the comparison by sex, which suggests that the factorial structure is equivalent between men and women. Next, the metric model (M1), which imposes constraints on factor loadings, was evaluated. The changes in fit indices were minimal compared to the configural model, supporting metric invariance. Subsequently, the scalar model (M2), which includes constraints on thresholds, was analyzed. The results continued to be satisfactory, allowing for the assumption of comparability of latent means between groups. Finally, the strict model (M3), which adds constraints on error variances, also showed a good fit, confirming strict invariance by sex.

**Table 5:** Measurement invariance.

Variable/model	WLSMV-χ^2^ (df)	RMSEA	CFI	TLI	SRMR	ΔRMSEA	ΔCFI	ΔTLI	Decision
Sex									
M0	76.166 (48)	0.054	0.995	0.992	0.021	–	–	–	Accepted
M1	90.803 (57)	0.054	0.993	0.992	0.028	0	−0.002	0	Accepted
M2	139.357 (96)	0.047	0.992	0.994	0.025	−0.007	−0.001	0.002	Accepted
M3	161.475 (105)	0.051	0.989	0.993	0.029	0.004	−0.003	−0.001	Accepted
Age									
M0	182.345 (66)	0.072	0.981	0.975	0.063	–	–	–	Accepted
M1	210.555 (75)	0.093	0.978	0.974	0.066	0.021	−0.003	−0.001	Rejected

WLSMV-χ^2^, Weighted Least Squares Mean and Variance adjusted chi-square; df, degrees of freedom; RMSEA, Root Mean Square Error of Approximation; CFI, Comparative Fit Index; TLI, Tucker–Lewis Index; SRMR, Standardized Root Mean Square Residual; Δ = change from the less constrained model.

In contrast, when evaluating invariance by age group, although the configural model (M0) showed acceptable fit, the metric model (M1) revealed a significant increase in RMSEA, exceeding the recommended cutoff values (Chen, 2007). Although changes in CFI and TLI were small, the deterioration in overall fit suggests that metric invariance is not met across age groups. This prevents further evaluation of more restrictive models and limits the comparison of structural parameters between these groups.

### 3.5 Convergent validity

Once the factorial structure of the scale was confirmed, convergent validity was assessed through the analysis of correlations between the three factors of the LWA-9 and the two dimensions of the SDO7 (Table 6). The SDO7 scale showed adequate internal consistency (Anti-egalitarianism *α* = 0.716, *ω* = 0.744; Dominance *α* = 0.956, *ω* = 0.965) and acceptable levels of validity [WLSMV-*χ*^2^(103) = 772.596, *p* < 0.001; CFI = 0.974; TLI = 0.970; RMSEA = 0.073 (90% CI = 0.069–0.077)]. The results show positive and statistically significant associations between both constructs, which are more pronounced in the case of the anti-egalitarianism dimension. These findings support the convergent validity of the LWA-9, as they are consistent with the theoretical expectation that a higher left-wing authoritarian orientation is related to lower adherence to social equality and lower acceptance of group hierarchy.

**Table 6:** Correlations between the factors of the LWA-9 and SDO scales.

Correlation between factors	*r*	*p*-value
Anti-hierarchical aggression with		
Anti-egalitarianism	0.231	*p* < 0.001
Dominance	0.168	*p* < 0.001
Anti-conventionalism with		
Anti-egalitarianism	0.392	*p* < 0.001
Dominance	0.260	*p* < 0.001
Top-down censorship with		
Anti-egalitarianism	0.341	*p* < 0.001
Dominance	0.283	*p* < 0.001

r = Pearson’s correlation coefficient; *p*-value = probability associated with the significance test.

### 3.6 Discriminant validity

To evaluate the discriminant validity of the LWA-9, bivariate correlations were calculated between its three dimensions (Anti-Hierarchical Aggression, Anti-Conventionalism, and Top-Down Censorship) and the total scores and three factors of the RWA scale. The results (Table 7) show that the correlations between the LWA-9 and RWA were low (*r* values ranging from −0.115 to 0.043) and mostly non-significant, suggesting that both scales measure distinct ideological constructs. Specifically, two correlations reached statistical significance: one between LWA-AHA and RWA-Total (*r* = −0.106, *p* < 0.05), and another between LWA-AC and RWA-CO (*r* = −0.115, *p* < 0.05). However, these correlations were weak in magnitude, reinforcing the conceptual independence of the two instruments within the analyzed sample.

**Table 7:** Bivariate correlations between global scores and dimensions of LWA-9 and RWA.

RWA Dimensions	LWA-AHA	LWA-AC	LWA-TDC
RWA-total	−0.106* (ns)	−0.039 (ns)	−0.094 (ns)
RWA-AA	−0.070 (ns)	−0.026 (ns)	−0.073 (ns)
RWA-SA	−0.041 (ns)	0.043 (ns)	−0.012 (ns)
RWA-CO	−0.090 (ns)	−0.115*	−0.092 (ns)

LWA, left-wing authoritarianism; AHA, anti-hierarchical aggression; AC, anti-conventionalism; TDC, top-down censorship; RWA, right-wing authoritarianism; AA, authoritarian aggression; SA, authoritarian submission; CO, conventionalism. Correlations were calculated using Pearson’s coefficient. *p < 0.05; ns, non-significant.

### 3.7 Evidence of reliability

Table 8 displays the reliability evidence for the LWA-9 scale based on the three-factor correlated model. The results show satisfactory to high internal consistency across all three factors, as assessed by McDonald’s omega (ω), Cronbach’s alpha (α), and the Greatest Lower Bound (GLB). Notably, the Anti-Conventionalism factor in the LWA-9 version demonstrated the highest reliability (GLB = 0.941; *ω* = 0.914; *α* = 0.913). In general, the abbreviated 9-item version (LWA-9) achieved comparable or superior reliability coefficients relative to the longer versions (LWA-39, LWA-25, LWA-13), particularly for Anti-Conventionalism and Top-Down Censorship. These findings provide support for the internal consistency of the LWA-9 across all dimensions.

**Table 8:** Comparative reliability of the left-wing authoritarianism factors across scale versions.

Factors	Scale version	McDonald’s *ω*	Cronbach’s *α*	GLB
Anti-hierarchical aggression	LWA-39	0.895	0.891	0.934
	LWA-25	0.896	0.895	0.931
	LWA-13	0.803	0.800	0.834
	LWA-9	0.861	0.852	0.883
Anti-conventionalism	LWA-39	0.919	0.917	0.936
	LWA-25	0.856	0.853	0.859
	LWA-13	0.784	0.782	0.790
	LWA-9	0.914	0.913	0.941
Top-down censorship	LWA-39	0.820	0.815	0.885
	LWA-25	0.734	0.732	0.819
	LWA-13	0.515	0.512	0.609
	LWA-9	0.825	0.824	0.873

GLB, greatest lower bound; ω, McDonald’s Omega; α, Cronbach’s Alpha. Reliability estimates are presented for each factor of the Left-Wing Authoritarianism Scale across its different versions.

## 4 Discussion

The present study aimed to evaluate the validity and reliability of three versions of the Left-Wing Authoritarianism Scale (Costello and Patrick, 2023) among Chilean university students and to propose a shortened 9-item version (LWA-9), which significantly reduces the instrument’s length and facilitates its use in applied contexts. These results indicate that all versions of the LWA demonstrated good psychometric indicators, confirming their adequacy and relevance for measuring left-wing authoritarianism in Chile. Moreover, the findings provide strong empirical evidence regarding the validity of this abbreviated version of the Left-Wing Authoritarianism Scale (LWA-9), which preserved a three-factor structure composed of the dimensions Anti-Hierarchical Aggression, Anti-Conventionalism, and Top-Down Censorship. In addition, the LWA-9 demonstrated superior factorial fit compared to the longer versions, greater efficiency, and showed adequate levels of internal reliability. These results are consistent with the original study, which highlights the relevance of the multidimensional construct of left-wing authoritarianism (Costello and Patrick, 2023). Consequently, this scale contributes to developing more suitable instruments for measuring authoritarianism and overcoming research biases (Duckitt, 2022; Fasce and Avendaño, 2022; García-Sánchez et al., 2022a,b).

Despite the significant reduction in the number of items, the results confirmed the stability and consistency of the factorial structure of the LWA, reinforcing its usefulness as a valid and efficient tool for measuring left-wing authoritarianism in Spanish-speaking contexts. It is worth noting that although the version used had been previously adapted into Spanish (Avendaño et al., 2022), it was deemed relevant to conduct a complementary linguistic evaluation in order to explore possible variations attributable to contextual and cultural differences in the Chilean case, specifically regarding the use of terms and expressions that could alter the understanding or interpretation of the items (Muñiz et al., 2013; Freiberg-Hoffmann et al., 2022). These can be found in the Supplementary material. In this regard, the abbreviated LWA-9 version represents a 31% reduction in the number of items compared to the LWA-13, while maintaining a clear factorial structure and solid psychometric properties. This shorter version enhances efficiency in contexts with time constraints or high measurement load, without compromising theoretical validity or the representativeness of the factors. This confirms the stability and coherence of the LWA’s factorial structure, reinforcing its role as a valid and practical tool for assessing left-wing authoritarianism in Spanish-speaking populations.

The confirmatory factor analysis supported the adequacy of the three-correlated-factor model, showing satisfactory fit indices. These results suggest that the proposed factorial structure optimally represents the underlying dimensions of the construct, providing a solid theoretical and methodological framework for its measurement. This structure’s consistency supports the instrument’s cross-cultural validity, although it is important to consider the particularities of each context (Avendaño et al., 2022).

Regarding convergent validity, the results show that the three factors of the LWA-9 scale are positively and statistically significantly correlated with both dimensions of the SDO7, especially with Anti-egalitarianism. These associations empirically support the convergent validity of the instrument, as they are consistent with the theoretical assumption that a higher left-wing authoritarian orientation is related to lower acceptance of social inequality and group hierarchy. In particular, the Anti-Conventionalism factor showed the strongest correlations, suggesting that this dimension is especially sensitive to beliefs about social equality. Moreover, these relationships reflect the contextual variability in the manifestation of the relationship between LWA and SDO (Costello et al., 2022; Milfont and Osborne, 2024; Peng, 2022; Van Hiel et al., 2024).

Furthermore, the discriminant validity of the LWA-9 was supported by the low magnitude and the lack of statistical significance in most bivariate correlations between its dimensions and those of the RWA. This pattern suggests that the LWA-9 measures a construct that is empirically distinct from right-wing authoritarianism. Although two correlations reached statistical significance, both were of weak magnitude, reinforcing the conceptual independence between left-wing and right-wing authoritarianism in this sample (Costello and Patrick, 2023; García-Sánchez et al., 2022a,b). Nevertheless, it is recommended that future studies continue to evaluate convergent and discriminant validity in more diverse samples and using other ideological constructs to further strengthen the functioning of the scale.

Regarding the evidence of reliability, the results show high coefficients for the three factors of the LWA scale model, indicating adequate internal consistency. Specifically, the values of McDonald’s *ω*, Cronbach’s *α*, and GLB are above the recommended threshold of 0.80 in every case. The Anti-Conventionalism factor stood out with the highest reliability indices, with a GLB of 0.941, followed by Anti-Hierarchical Aggression and Top-Down Censorship, whose values also reflect high reliability. These results support the stability and precision of the measurement of each dimension of the scale.

In this sense, the present study offers rigorous empirical evidence on the factorial structure, convergent validity, and reliability of the abbreviated version of the Left-Wing Authoritarianism Scale (LWA-9) in a sample of university students, establishing itself as a psychometrically robust tool for evaluating progressive authoritarian attitudes, which include Anti-Hierarchical Aggression, Anti-Conventionalism, and Top-Down Censorship. In this regard, the study’s results provide a more accurate assessment of authoritarianism in the Chilean context, taking into account sociohistorical conditions, and also contribute to clarifying the general nature of the construct (Costello et al., 2022; Duckitt et al., 2010; Obreque et al., 2024).

A relevant methodological aspect of the present research is the thorough evaluation of the factorial invariance of the LWA-9 across sociodemographic groups. Our multigroup analyses demonstrated strict invariance by sex, which implies that the factorial structure, loadings, thresholds, and measurement errors are equivalent between men and women. This ensures that mean comparisons between both groups are valid and not biased by differences in item interpretation or functioning, representing a significant methodological advancement in assessing LWA invariance for psychological studies (Nilsson, 2024) compared to previous literature (Costello and Patrick, 2023; Avendaño et al., 2022; Krispenz et al., 2025).

However, metric invariance was not achieved in the analyses by age group. This suggests that the scale may not function entirely equivalently across different age ranges. This result contrasts with that of Lambert et al. (2024), who reported scalar invariance by age. Therefore, comparisons between these groups should be interpreted with caution, as they may reflect possible sample or cultural effects. These findings reinforce the robustness of the LWA-9 for gender-based studies but also highlight the need for future research to further explore the measurement equivalence across other sociodemographic variables.

It should also be noted that the sampling design constitutes a limitation of the study, as a non-probabilistic convenience sampling method was used, with an overrepresentation of women and students from the social sciences. Although the results obtained are robust, they should also be considered preliminary, given that this sample configuration restricts the ability to generalize the findings to other cultural contexts and more diverse university populations. As Hernández-Sampieri et al. (2014) point out, this type of sampling—although common in applied research—reduces representativeness and limits the extrapolation of results. Therefore, future research is encouraged to replicate the study with more heterogeneous samples, greater disciplinary diversity, and probabilistic sampling strategies.

Another limitation concerns the evaluation of the factorial invariance of the LWA-9 according to participants’ political orientation. Although a considerable proportion of individuals identified with left-wing and center-left positions (34.7%), the distribution of the remaining categories was markedly uneven, with a majority not identifying with any political position (44.4%) and low representation of center-right and right-wing (15.6%) and center (5.3%) orientations. Given that the WLSMV estimator used in multigroup models requires relatively balanced sample sizes per group to ensure the stability of the estimates, this distribution limited the robust analysis of factor invariance according to political orientation. In this regard, it is suggested that future research address this issue through more balanced sampling. Despite these limitations, the LWA-9 is beneficial for researchers seeking to expand the assessment of authoritarian attitudes, especially in contexts that require the efficient administration of multiple instruments.

Another possible limitation of this study relates to the conceptual differentiation between some of the evaluated dimensions, specifically between Anti-Conventionalism and Top-Down Censorship. Although the assumed theoretical model distinguishes between these constructs, their semantic proximity may reflect an overlap in attitudinal content, making empirical discrimination between them more difficult. This aspect highlights the need for future research to revisit the conceptual clarity of these dimensions and to explore alternative models or item reformulations that more accurately capture their distinct characteristics.

The abbreviated version of the LWA-9 provides a comprehensive measurement of authoritarian attitudes within the progressive sphere, establishing itself as a theoretically sound construct that enhances its applicability in educational contexts. This allows for a broader analysis of authoritarianism from left-wing ideological perspectives—an approach that has been subject to controversy—and helps overcome the widespread exclusion of leftist ideological traits in historical conceptualizations of the phenomenon (Conway et al., 2018; Costello et al., 2022; Malecki et al., 2025; Manson, 2020).

Additionally, it is beneficial for researchers who need to administer multiple instruments simultaneously, as it reduces respondent burden and significantly decreases the need for resources. Its usefulness aligns with the need to develop efficient measurement tools to understand current sociopolitical dynamics (Campbell and Manning, 2018; Lukianoff and Haidt, 2019) and to identify its presence in Spanish-speaking contexts (Fasce and Avendaño, 2022). The LWA-9 represents a solid contribution to expanding the discussion within the international literature without compromising its theoretical validity or the representativeness of its factors.

## 5 Conclusion

The present study’s findings confirm that the abbreviated version of the Left-Wing Authoritarianism Scale (LWA) presents adequate indicators of validity and reliability among Chilean university students. This abbreviated version represents a robust methodological alternative for studies requiring the simultaneous evaluation of multiple constructs, optimizing administration time without compromising the validity and reliability of the measurement.

The scale allows for the capture of authoritarian attitudes associated with progressive ideologies, offering a complementary approach to traditional studies focused on right-wing authoritarianism. Its application is particularly relevant in educational contexts, where it can contribute to analyzing ideological polarization, political intolerance, institutional climate, and democratic coexistence.

About the above, its use opens possibilities for the design of interventions aimed at strengthening democratic coexistence, critical thinking, and respect for ideological diversity in university settings. These results support the need to continue developing and adapting instruments that are sensitive to the Latin American sociopolitical context, promoting a more comprehensive understanding of authoritarianism among young populations.

In this sense, the present study provides valuable empirical evidence supporting the psychometric robustness of the LWA among university students in Chile, reinforcing its usefulness as a diagnostic and research tool in educational contexts.

## Data Availability

The raw data supporting the conclusions of this article will be made available by the authors, without undue reservation.
