# Supplementary material for: Psychometric study and validation of an abbreviated version of the Left-Wing Authoritarianism Scale (LWA-9) in Chilean university students
Source: Front Psychol. 2025 Aug 29;16:1627540. doi: 10.3389/fpsyg.2025.1627540 (PMC12426141; doi:10.3389/fpsyg.2025.1627540)
Supplement: Supplementary file 1 [file Data_Sheet_1.pdf]

Supplementary material / Material suplementario

1. Left-Wing Authoritarianism Index (LWA) used in the present study. / Escala de Autoritarismo de Izquierda (LWA) utilizada en el estudio actual.

Left-Wing Authoritarianism Index LWA (Costello & Patrick, 2023)

Please indicate the extent to which you agree or disagree with each of the following statements by selecting the option that best represents your opinion.

		Strongly Disapprove	Disapprove	Slightly Disapprove	Neither Approve nor [illegible]	Slightly Approve	Approve	Strongly Approve
1	**The rich should be stripped of their belongings and status. (*)**	☐	☐	☐	☐	☐	☐	☐
2	**Rich people should be forced to give up virtually all of their wealth. (*)**	☐	☐	☐	☐	☐	☐	☐
3	**If I could remake society, I would put people who currently have the most privilege at the very bottom. (*)**	☐	☐	☐	☐	☐	☐	☐
4	America would be much better off if all of the rich people were at the bottom of the social ladder.	☐	☐	☐	☐	☐	☐	☐
5	When the tables are turned on the oppressors at the top of society, I will enjoy watching them suffer the violence that they have inflicted on so many others.	☐	☐	☐	☐	☐	☐	☐
6	Most rich Wall Street executives deserve to be thrown in prison.	☐	☐	☐	☐	☐	☐	☐
7	Constitutions and laws are just another way for the powerful to destroy our dignity and individuality.	☐	☐	☐	☐	☐	☐	☐
8	The current system is beyond repair.	☐	☐	☐	☐	☐	☐	☐
9	We need to replace the established order by any means necessary.	☐	☐	☐	☐	☐	☐	☐

10	Political violence can be constructive when it serves the cause of social justice.	☐	☐	☐	☐	☐	☐	☐
11	Certain elements in our society must be made to pay for the violence of their ancestors.	☐	☐	☐	☐	☐	☐	☐
12	If a few of the worst Republican politicians were assassinated, it wouldn't be the end of the world.	☐	☐	☐	☐	☐	☐	☐
13	I would prefer a far-left leader with absolute authority over a right-wing leader with limited power.	☐	☐	☐	☐	☐	☐	☐
14	Schools should be required by law to teach children about our country's history of racism, classism, sexism, and homophobia.	☐	☐	☐	☐	☐	☐	☐
15	Anyone who opposes gay marriage must be homophobic.	☐	☐	☐	☐	☐	☐	☐
16	**Deep down, just about all conservatives are racist, sexist, and homophobic. (*)**	☐	☐	☐	☐	☐	☐	☐
17	**People who are truly worried about terrorism should shift their focus to the nutjobs on the far-right. (*)**	☐	☐	☐	☐	☐	☐	☐
18	The "old-fashioned ways" and "old-fashioned values" need to be abolished.	☐	☐	☐	☐	☐	☐	☐
19	Radical and progressive moral values can save our society.	☐	☐	☐	☐	☐	☐	☐
20	**All political conservatives are fools. (*)**	☐	☐	☐	☐	☐	☐	☐
21	I cannot imagine myself becoming friends with a political conservative.	☐	☐	☐	☐	☐	☐	☐
22	Conservatives are morally inferior to liberals.	☐	☐	☐	☐	☐	☐	☐
23	It is important that we destroy the West's nationalist, imperialist values.	☐	☐	☐	☐	☐	☐	☐
24	I try to expose myself to conservative news sources.	☐	☐	☐	☐	☐	☐	☐

25	There is nothing wrong with Bible camps.	☐	☐	☐	☐	☐	☐	☐
26	I hate being around non-progressive people.	☐	☐	☐	☐	☐	☐	☐
27	Classroom discussions should be safe places that protect students from disturbing ideas.	☐	☐	☐	☐	☐	☐	☐
28	University authorities are right to ban hateful speech from campus.	☐	☐	☐	☐	☐	☐	☐
29	I should have the right not to be exposed to offensive views.	☐	☐	☐	☐	☐	☐	☐
30	To succeed, a workplace must ensure that its employees feel safe from criticism.	☐	☐	☐	☐	☐	☐	☐
31	We must line up behind strong leaders who have the will to stamp out prejudice and intolerance.	☐	☐	☐	☐	☐	☐	☐
32	When we spend all of our time protecting the right to "free speech" we're protecting the rights of sexists, racists, and homophobes at the cost of marginalized people.	☐	☐	☐	☐	☐	☐	☐
33	**I am in favor of allowing the government to shut down right-wing internet sites and blogs that promote nutty, hateful positions. (*)**	☐	☐	☐	☐	☐	☐	☐
34	**Colleges and universities that permit speakers with intolerant views should be publicly condemned. (*)**	☐	☐	☐	☐	☐	☐	☐
35	Getting rid of inequality is more important than protecting the so-called "right" to free speech.	☐	☐	☐	☐	☐	☐	☐
36	**Fox News, right-wing talk radio, and other conservative media outlets should be prohibited from broadcasting their hateful views. (*)**	☐	☐	☐	☐	☐	☐	☐
37	Even books that contain racism or racial language should not be censored.	☐	☐	☐	☐	☐	☐	☐
38	I don't support shutting down speakers with sexist, homophobic, or racist views.	☐	☐	☐	☐	☐	☐	☐
39	Neo-Nazis ought to have a legal right to their opinions.	☐	☐	☐	☐	☐	☐	☐

Notes:
-The items marked with an asterisk (*) and in bold correspond to the abbreviated 9-item version of the scale.

**Escala de Autoritarismo de Izquierda (LWA, por sus siglas en inglés) (Costello & Patrick, 2023)**								
Por favor, indique en qué medida está de acuerdo o en desacuerdo con cada una de las siguientes afirmaciones seleccionando la opción que mejor represente su opinión.								
Ítems		Strongly Disapprove	Disapprove	Slightly Disapprove	Neither Approve nor [illegible]	Slightly Approve	Approve	Strongly Approve
1	**Las personas con mayor riqueza económica deben ser despojadas de sus pertenencias y estatus social. (*)**	☐	☐	☐	☐	☐	☐	☐
2	**Las personas con mayor riqueza deberían ser forzadas a renunciar a toda su riqueza. (*)**	☐	☐	☐	☐	☐	☐	☐
3	**Si pudiera reordenar la sociedad, pondría en la parte más baja de la sociedad a las personas que actualmente tienen los mayores privilegios. (*)**	☐	☐	☐	☐	☐	☐	☐
4	Mi país estaría mucho mejor si todas las personas ricas estuvieran al fondo en la escala social	☐	☐	☐	☐	☐	☐	☐
5	Cuando cambien los privilegios de los opresores situados en la cima de la sociedad, disfrutaré viéndolos sufrir la violencia que ellos mismos han infligido a tantos otros.	☐	☐	☐	☐	☐	☐	☐
6	La mayoría de los grandes empresarios nacionales merecen ser encarcelados.	☐	☐	☐	☐	☐	☐	☐
7	Las constituciones y las leyes son sólo otra forma para que los poderosos destruyan nuestra dignidad e individualidad.	☐	☐	☐	☐	☐	☐	☐
8	El sistema actual no tiene solución.	☐	☐	☐	☐	☐	☐	☐
9	Necesitamos reemplazar el orden establecido a través de cualquier medio necesario.	☐	☐	☐	☐	☐	☐	☐

10	La violencia política puede ser constructiva cuando sirve a la causa de la justicia social.	☐	☐	☐	☐	☐	☐	☐
11	Algunas personas de nuestra sociedad deben pagar por la violencia ejercida por sus antepasados.	☐	☐	☐	☐	☐	☐	☐
12	No sería tan grabe para la sociedad si algunos de los peores políticos de derechas fueran asesinados.	☐	☐	☐	☐	☐	☐	☐
13	Preferiría un líder de extrema izquierda con autoridad absoluta antes que un líder de derecha con poder limitado.	☐	☐	☐	☐	☐	☐	☐
14	La ley debería exigir a las escuelas enseñar a las y los niños sobre nuestra historia de racismo, clasismo, machismo y homofobia.	☐	☐	☐	☐	☐	☐	☐
15	Todos aquellos que se oponen al matrimonio homosexual son homofóbicos.	☐	☐	☐	☐	☐	☐	☐
16	**En el fondo, casi todas las personas de derechas son racistas, sexistas y homofóbicas. (*)**	☐	☐	☐	☐	☐	☐	☐
17	**Aquellos que están tan preocupados por el terrorismo deberían prestar más atención a los payasos de extrema derecha a los que apoyan. (*)**	☐	☐	☐	☐	☐	☐	☐
18	Las "formas y valores tradicionales" deben ser erradicados.	☐	☐	☐	☐	☐	☐	☐
19	Los valores morales radicalmente progresistas pueden salvar nuestra sociedad.	☐	☐	☐	☐	☐	☐	☐
20	**La gente de derecha es imbécil. (*)**	☐	☐	☐	☐	☐	☐	☐
21	No me imagino siendo amigo de una persona de derecha.	☐	☐	☐	☐	☐	☐	☐
22	La gente de derecha es moralmente inferior a la gente de izquierda.	☐	☐	☐	☐	☐	☐	☐
23	Es importante que destruyamos los valores nacionalistas e imperialistas de occidente.	☐	☐	☐	☐	☐	☐	☐
24	Suelo escuchar a periodistas y medios de comunicación conservadores	☐	☐	☐	☐	☐	☐	☐

25	No hay nada malo en los campamentos y retiros cristianos para niños	☐	☐	☐	☐	☐	☐	☐
26	Me desagrada estar rodeado de personas conservadoras.	☐	☐	☐	☐	☐	☐	☐
27	Las aulas de los centros de enseñanza deberían ser lugares seguros que protejan a los estudiantes de ideas perturbadoras.	☐	☐	☐	☐	☐	☐	☐
28	Las autoridades universitarias tienen razón al prohibir discursos de odio en el campus.	☐	☐	☐	☐	☐	☐	☐
29	Debería tener el derecho a no ser expuesto a puntos de vista ofensivos.	☐	☐	☐	☐	☐	☐	☐
30	Para tener éxito, el entorno laboral debe garantizar que los empleados se sientan a salvo de la crítica.	☐	☐	☐	☐	☐	☐	☐
31	Debemos apoyar a líderes fuertes que tengan la voluntad de acabar con los prejuicios y la intolerancia.	☐	☐	☐	☐	☐	☐	☐
32	Cuando protegemos la "libertad de expresión", estamos defendiendo los derechos de los machistas, racistas y homofóbicos a costa de las personas oprimidas.	☐	☐	☐	☐	☐	☐	☐
33	**Estoy a favor de permitir que el gobierno cierre páginas y blogs de derecha que promuevan ideas locas y llenas de odio. (*)**	☐	☐	☐	☐	☐	☐	☐
34	**Los colegios y universidades que permiten oradores que defienden opiniones intolerantes deben ser condenados públicamente. (*)**	☐	☐	☐	☐	☐	☐	☐
35	Acabar con la desigualdad es más importante que defender el supuesto "derecho" a la libertad de expresión.	☐	☐	☐	☐	☐	☐	☐
36	**Los programas de televisión, radio y otros medios de comunicación de derechas deberían tener prohibido retransmitir sus puntos de vista intolerantes. (*)**	☐	☐	☐	☐	☐	☐	☐
37	Los libros que promueven el racismo o contienen lenguaje racista no deberían ser censurados	☐	☐	☐	☐	☐	☐	☐
38	No apoyo que se censure a aquellos que tienen puntos de vista machistas, homófobos o racistas	☐	☐	☐	☐	☐	☐	☐

39	Los neonazis deberían tener derecho a defender sus opiniones	☐	☐	☐	☐	☐	☐	☐
Notas:<br><br>- Los ítems señalados con un asterisco (*) y en negrita corresponden a la versión abreviada de 9 ítems de la escala.								

2. Comparative Table of the Left-Wing Authoritarianism Index Items in the Original, Spanish, and Chilean Versions/ Tabla comparativa de los ítems del Índice de Autoritarismo de Izquierda en sus versiones original, española y chilena

**Ítems**	**Original scale in English (Costello & Patrick, 2023)**	**Version adapted into Spanish (Avendaño et al., 2022)**	**Linguistic adaptation Chilean version**
1	**The rich should be stripped of their belongings and status. (*)**	Los ricos deben ser despojados de sus pertenencias y estatus.	**(Las personas con mayor riqueza económica) deben ser despojadas de sus pertenencias y estatus (social). (*)**
2	**Rich people should be forced to give up virtually all of their wealth. (*)**	Los ricos deberían ser forzados a renunciar a prácticamente toda su riqueza.	**(Las personas con mayor riqueza) deberían ser forzadas a renunciar a (eliminando: prácticamente) toda su riqueza. (*)**
3	**If I could remake society, I would put people who currently have the most privilege at the very bottom. (*)**	Si pudiera rehacer la sociedad, pondría en lo más bajo a las personas que actualmente tienen los mayores privilegios.	**Si pudiera (reordenar) la sociedad, pondría en (la parte más baja de la sociedad) a las personas que actualmente tienen los mayores privilegios. (*)**
4	America would be much better off if all of the rich people were at the bottom of the social ladder.	Mi país estaría mucho mejor si todas las personas ricas estuvieran al final de la escala social.	Mi país estaría mucho mejor si todas las personas ricas estuvieran al (fondo en) la escala social
5	When the tables are turned on the oppressors at the top of society, I will enjoy watching them suffer the violence that they have inflicted on so many others.	Cuando cambien las tornas respecto a los opresores situados en la cima de la sociedad, disfrutaré viéndolos sufrir la violencia que ellos	Cuando cambien (los privilegios de) los opresores situados en la cima de la sociedad, disfrutaré viéndolos sufrir la violencia que ellos mismos han infligido a tantos otros.

		mismos han infligido a tantos otros.	
6	Most rich Wall Street executives deserve to be thrown in prison.	La mayoría de los ejecutivos ricos de Wall Street merecen ser encarcelados.	La mayoría de los (grandes empresarios nacionales) merecen ser encarcelados.
7	Constitutions and laws are just another way for the powerful to destroy our dignity and individuality.	Las constituciones y las leyes son sólo otra forma para que los poderosos destruyan nuestra dignidad e individualidad.	Las constituciones y las leyes son sólo otra forma para que los poderosos destruyan nuestra dignidad e individualidad.
8	The current system is beyond repair.	El sistema actual es irreparable.	El sistema actual (no tiene solución).
9	We need to replace the established order by any means necessary.	Necesitamos reemplazar el orden establecido a través de cualquier medio necesario.	Necesitamos reemplazar el orden establecido a través de cualquier medio necesario.
10	Political violence can be constructive when it serves the cause of social justice.	La violencia política puede ser constructiva cuando sirve a la causa de la justicia social.	La violencia política puede ser constructiva cuando sirve a la causa de la justicia social.
11	Certain elements in our society must be made to pay for the violence of their ancestors.	Ciertas personas de nuestra sociedad deben pagar por la violencia ejercida por sus antepasados.	(Algunas) personas de nuestra sociedad deben pagar por la violencia ejercida por sus antepasados.
12	If a few of the worst Republican politicians were assassinated, it wouldn't be the end of the world.	No sería para tanto si algunos de los peores políticos de derechas fueran asesinados.	No sería (tan grabe para la sociedad) si algunos de los peores políticos de derechas fueran asesinados.
13	I would prefer a far-left leader with absolute authority over a right-wing leader with limited power.	Preferiría un líder de extrema izquierda con autoridad absoluta antes que un líder de derechas con poder limitado.	Preferiría un líder de extrema izquierda con autoridad absoluta antes que un líder de derecha con poder limitado.
14	Schools should be required by law to teach children about our country's history of racism, classism, sexism, and homophobia.	La ley debería exigir a las escuelas enseñar a los niños sobre nuestra historia de racismo, clasismo, machismo y homofobia.	La ley debería exigir a las escuelas enseñar a las y los niños sobre nuestra historia de racismo, clasismo, machismo y homofobia.
15	Anyone who opposes gay marriage must be homophobic.	Todos aquellos que se oponen al matrimonio homosexual son homófobos.	Todos aquellos que se oponen al matrimonio homosexual son (homofóbicos).
16	**Deep down, just about all conservatives are racist, sexist, and homophobic. (*)**	En el fondo, casi todas las personas de derechas son racistas, sexistas y homófobas.	**En el fondo, casi todas las personas de derechas son racistas, sexistas y (homofóbicas). (*)**
17	**People who are truly worried about terrorism should shift their focus to the nutjobs on the far-right. (*)**	Aquellos que están tan preocupados por el terrorismo deberían prestar más atención a los chiflados de extrema derecha a los que apoyan.	**Aquellos que están tan preocupados por el terrorismo deberían prestar más atención a los (payasos) de extrema derecha a los que apoyan. (*)**
18	The "old-fashioned ways" and "old-fashioned values" need to be abolished.	Las “formas y valores tradicionales” deben ser abolidos.	Las “formas y valores tradicionales” deben ser (erradicados).
19	Radical and progressive moral values can save our society.	Los valores morales radicalmente progresistas pueden salvar nuestra sociedad.	Los valores morales radicalmente progresistas pueden salvar nuestra sociedad.
20	**All political conservatives are fools. (*)**	La gente de derechas es imbécil.	**La gente de derecha es imbécil. (*)**

21	I cannot imagine myself becoming friends with a political conservative.	No me imagino siendo amigo de una persona de derechas.	No me imagino siendo amigo de una persona de (derecha).
22	Conservatives are morally inferior to liberals.	La derecha es moralmente inferior a la izquierda.	La (gente de) derecha es moralmente inferior a la (gente de) izquierda.
23	It is important that we destroy the West's nationalist, imperialist values.	Es importante que destruyamos los valores nacionalistas e imperialistas de occidente.	Es importante que destruyamos los valores nacionalistas e imperialistas de occidente.
24	I try to expose myself to conservative news sources.	Suelo escuchar a periodistas y medios de comunicación conservadores (R).	Suelo escuchar a periodistas y medios de comunicación conservadores (R).
25	There is nothing wrong with Bible camps.	No hay nada malo en los campamentos y retiros cristianos para niños (R).	No hay nada malo en los campamentos y retiros cristianos para niños (R).
26	I hate being around non-progressive people.	Odio estar rodeado de personas conservadoras.	(Me desagrada) estar rodeado de personas conservadoras.
27	Classroom discussions should be safe places that protect students from disturbing ideas.	Las aulas de los centros de enseñanza deberían ser lugares seguros que protejan a los estudiantes de ideas perturbadoras.	Las aulas de los centros de enseñanza deberían ser lugares seguros que protejan a los estudiantes de ideas perturbadoras.
28	University authorities are right to ban hateful speech from campus.	Las autoridades universitarias tienen razón al prohibir discursos de odio en el campus.	Las autoridades universitarias tienen razón al prohibir discursos de odio en el campus.
29	I should have the right not to be exposed to offensive views.	Debería tener el derecho a no ser expuesto a puntos de vista ofensivos.	Debería tener el derecho a no ser expuesto a puntos de vista ofensivos.
30	To succeed, a workplace must ensure that its employees feel safe from criticism.	Para tener éxito, el entorno laboral debe garantizar que los empleados se sientan a salvo de la crítica.	Para tener éxito, el entorno laboral debe garantizar que los empleados se sientan a salvo de la crítica.
31	We must line up behind strong leaders who have the will to stamp out prejudice and intolerance.	Debemos apoyar a lideres fuertes que tengan la voluntad de acabar con los prejuicios y la intolerancia.	Debemos apoyar a líderes fuertes que tengan la voluntad de acabar con los prejuicios y la intolerancia.
32	When we spend all of our time protecting the right to "free speech" we're protecting the rights of sexists, racists, and homophobes at the cost of marginalized people.	Cuando protegemos la "libertad de expresión," estamos defendiendo los derechos de los machistas, racistas y homófobos a costa de las personas oprimidas.	Cuando protegemos la "libertad de expresión", estamos defendiendo los derechos de los machistas, racistas y (homofóbicos) a costa de las personas oprimidas.
33	**I am in favor of allowing the government to shut down right-wing internet sites and blogs that promote nutty, hateful positions. (*)**	Estoy a favor de permitir que el gobierno cierre páginas y blogs de derecha que promuevan ideas disparatadas y llenas de odio.	**Estoy a favor de permitir que el gobierno cierre páginas y blogs de derecha que promuevan ideas (locas) y llenas de odio. (*)**
34	**Colleges and universities that permit speakers with intolerant views should be publicly condemned. (*)**	Los colegios y universidades que permiten oradores que defienden opiniones intolerantes deben ser condenados públicamente.	**Los colegios y universidades que permiten oradores que defienden opiniones intolerantes deben ser condenados públicamente. (*)**

35	Getting rid of inequality is more important than protecting the so-called "right" to free speech.	Acabar con la desigualdad es más importante que defender el supuesto "derecho" a la libertad de expresión.	Acabar con la desigualdad es más importante que defender el supuesto "derecho" a la libertad de expresión.
36	**Fox News, right-wing talk radio, and other conservative media outlets should be prohibited from broadcasting their hateful views. (*)**	Los programas de televisión, radio y otros medios de comunicación de derechas deberían tener prohibido retransmitir sus puntos de vista intolerantes.	**Los programas de televisión, radio y otros medios de comunicación de derechas deberían tener prohibido retransmitir sus puntos de vista intolerantes. (*)**
37	Even books that contain racism or racial language should not be censored.	Los libros que contienen racismo o lenguaje racista no deberían ser censurados (R).	Los libros que (promueven el) racismo o (contienen) lenguaje racista no deberían ser censurados (R).
38	I don't support shutting down speakers with sexist, homophobic, or racist views.	No apoyo que se censure a aquellos que tienen puntos de vista machistas, homófobos o racistas (R).	No apoyo que se censure a aquellos que tienen puntos de vista machistas, homófobos o racistas (R).
39	Neo-Nazis ought to have a legal right to their opinions.	Los neonazis deberían tener derecho a defender sus opiniones (R).	Los neonazis deberían tener derecho a defender sus opiniones (R).

Note: Words or phrases in parentheses in the Chilean version column indicate linguistic adaptations relative to the previously validated Spanish version.

- When a word or expression has been modified, the new version appears in parentheses.
- When a word from the Spanish version has been removed in the Chilean version without replacement, this is indicated with the notation (removed: original word).
- Items marked with an asterisk (*) and in bold correspond to the abbreviated 9-item version of the scale.

Nota: Las palabras o frases entre paréntesis en la columna de la versión chilena indican adaptaciones lingüísticas respecto a la versión española previamente validada.

- Cuando se ha **modificado** una palabra o expresión, la nueva versión aparece entre paréntesis.
- Cuando una palabra de la versión española ha sido **eliminada** en la versión chilena sin ser reemplazada, se indica con la notación (eliminado: palabra original).
- Los ítems señalados con un asterisco (*) y en negrita corresponden a la versión abreviada de 9 ítems de la escala.
